# Rectal Swabs from Critically Ill Patients Provide Discordant Representations of the Gut Microbiome Compared to Stool Samples

**DOI:** 10.1128/mSphere.00358-19

**Published:** 2019-07-24

**Authors:** Katherine Fair, Daniel G. Dunlap, Adam Fitch, Tatiana Bogdanovich, Barbara Methé, Alison Morris, Bryan J. McVerry, Georgios D. Kitsios

**Affiliations:** a Division of Pulmonary, Allergy and Critical Care Medicine, Department of Medicine, University of Pittsburgh School of Medicine and University of Pittsburgh Medical Center, Pittsburgh, Pennsylvania, USA; b Center for Medicine and the Microbiome, University of Pittsburgh, Pittsburgh, Pennsylvania, USA; c Division of Infectious Diseases, University of Pittsburgh Medical Center, Pittsburgh, Pennsylvania, USA; d Department of Immunology, University of Pittsburgh School of Medicine, Pittsburgh, Pennsylvania, USA; The Jackson Laboratory for Genomic Medicine

**Keywords:** gut dysbiosis, gut microbiome, microbiome, rectal swab, stool

## Abstract

The role of the gut microbiome in critical illness is being actively investigated, but the optimal sampling methods for sequencing studies of gut microbiota remain unknown. Stool samples are generally considered the reference standard but are not practical to obtain in the intensive care unit (ICU), and thus, rectal swabs are often used. However, the reliability of rectal swabs for gut microbiome profiling has not been established in the ICU setting. In this study, we compared 16S rRNA gene sequencing results between rectal swab and stool samples collected at three time points from mechanically ventilated critically ill adults. Rectal swabs comprised 89% of the samples collected at the baseline time point, but stool samples became more extensively available at later time points. Significant differences in alpha-diversity and beta-diversity between rectal swabs and stool samples were observed, but these differences were primarily due to baseline samples. Higher relative abundances of members of the *Actinobacteria* phylum (typically skin microbes) were present in rectal swabs than in stool samples (*P* = 0.05), a difference that was attenuated over time. The progressively increasing similarity of rectal swabs and stool samples likely resulted from increasing levels of stool coating of the rectal vault and direct soiling of the rectal swabs taken at later time points. Therefore, inferences about the role of the gut microbiome in critical illness should be drawn cautiously and should take into account the type and timing of samples analyzed.

**IMPORTANCE** Rectal swabs have been proposed as potential alternatives to stool samples for gut microbiome profiling in outpatients or healthy adults, but their reliability in assessment of critically ill patients has not been defined. Because stool sampling is not practical and often not feasible in the intensive care unit, we performed a detailed comparison of gut microbial sequencing profiles between rectal swabs and stool samples in a longitudinal cohort of critically ill patients. We identified systematic differences in gut microbial profiles between rectal swabs and stool samples and demonstrated that the timing of the rectal swab sampling had a significant impact on sequencing results. Our methodological findings should provide valuable information for the design and interpretation of future investigations of the role of the gut microbiome in critical illness.

## Abstract (precis)

Rectal swabs have been proposed as potential alternatives to stool samples for gut microbiome profiling in outpatients or healthy adults, but their reliability in assessment of critically ill patients has not been defined. Because stool sampling is not practical and often not feasible in the intensive care unit, we performed a detailed comparison of gut microbial sequencing profiles between rectal swabs and stool samples in a longitudinal cohort of critically ill patients. We identified systematic differences in gut microbial profiles between rectal swabs and stool samples and demonstrated that the timing of the rectal swab sampling had a significant impact on sequencing results. Our methodological findings should provide valuable information for the design and interpretation of future investigations of the role of the gut microbiome in critical illness.

## OBSERVATION

Gut microbial dysbiosis is plausibly a contributor to the onset, evolution, and outcome of critical illness, but the mechanisms involved have not been fully elucidated (1, 2). Fecal microbial communities in critically ill adults display lower diversity than and distinct taxonomic signatures compared to fecal samples from healthy individuals (3). Thus, defining the pathogenetic disruptions of gut communities during critical illness may help identify new targets for intervention (1, 2).

Sampling gut microbiota for sequencing analyses can be challenging in the intensive care unit (ICU). Critically ill patients frequently experience constipation or ileus (4), and the provision of early enteral nutrition is highly variable (5). Thus, critically ill patients may not have any bowel movements, especially early in their ICU course. Furthermore, standard decontamination practices in ICU care (6) often result in stool disposal before samples are collected. For these reasons, rectal swab use represents an attractive, minimally invasive method for sampling gut microbiota, which is routinely used in clinical practice for screening of vancomycin-resistant *Enterococcus* colonization.

Rectal swabs have been proposed as potential alternatives to stool samples in ambulatory patients (7, 8), but data in critical illness are limited. Recent work from Bansal et al. (9) in analyses of nine critically ill patients showed compositional discrepancies between rectal swabs and stool samples when rectal swabs were not visibly soiled by stool. To further investigate systematic differences in the gut microbial profiles captured by stool specimens versus rectal swab samples, we obtained data from a larger cohort of 106 patients admitted to the medical ICU at a tertiary care academic center.

Detailed methods are reported in Text S1 in the supplemental material. Briefly, in this observational cohort study, we prospectively enrolled consecutive mechanically ventilated patients with acute respiratory failure from any cause. We collected rectal swabs and/or stool samples at baseline (days 0 to 2 from intubation) and at the middle (days 3 to 6) and late (days 7 to 10) intervals of follow-up starting at the time of intubation and continuing for up to 10 days if the patient remained in the ICU. Rectal swabs were collected according to a standard operating procedure (i.e., placing the patient in a lateral position, inserting the cotton tip of the swab into the rectal canal, and rotating the swab gently for 5 s), unless clinical reasons precluded movement of the patient (e.g., severe hemodynamic or respiratory instability). Stool samples were collected when available, either by taking a small sample from an expelled bowel movement (before cleaning of the patient and disposal of the stool) or from a fecal management system (rectal tube) placed for management of diarrhea and liquid stool collection. For comparisons with healthy gut microbiota, we also included 15 stool samples obtained from healthy volunteers used for fecal microbiota transplantation (FMT stool). We extracted bacterial DNA and performed 16S rRNA gene sequencing (V4 region) using an Illumina MiSeq system with standard protocols as previously described (10) and as detailed in Text S1. Sequencing data were analyzed for alpha/beta-diversity and taxonomic composition using R software. To assess longitudinal changes of alpha-diversity over time as well as to account for the effects of potential confounders on the associations between sample type and gut microbiota profiles, we constructed a set of multivariate models (Text S1), adjusting for the following potential confounders: (i) early enteral nutrition (initiation of gastric or enteral feeds within 48 h of ICU admission); (ii) severity of illness by Sequential Organ Failure Assessment (SOFA) scores; (iii) presence of rectal tube (indicator of severe diarrhea); (iv) obesity; (v) clinical diagnosis of sepsis.

10.1128/mSphere.00358-19.1 Text S1 Supplemental methods. Download Text S1, PDF file, 0.2 MB. Copyright © 2019 Fair et al. 2019 Fair et al. This content is distributed under the terms of the Creative Commons Attribution 4.0 International license.

**FIG 1:**
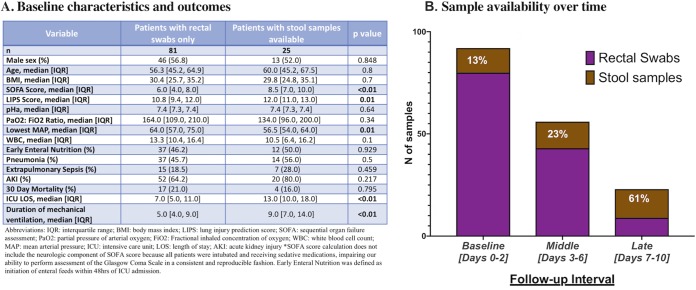
Cohort characteristics and sample type availability over time. (A) Table listing baseline characteristics and clinical outcomes of patients with rectal swabs only versus patients with stool samples available. *P* values are from Wilcoxon tests for continuous and Fisher’s exact tests for categorical variables (highlighted in bold when significant [*P* < 0.05]). (B) Stacked-bar graph of numbers of rectal swabs versus stool samples at each time interval (purple for rectal swabs and brown for stool samples). The proportion of stool samples available at each time interval is shown with white characters.

We enrolled 106 patients with a total of 171 samples (132 rectal swabs and 39 stool samples). Stool samples were available from 25 (24%) patients during the study period, and 10 patients had both sample types available. Patients with stool samples available had baseline demographics (age, sex, body mass index [BMI]) similar to those of the patients without stool samples (Fig. 1A) but had higher SOFA scores and longer duration of ICU stay and mechanical ventilation (Wilcoxon *P* < 0.001). Sample type availability varied by follow-up interval as follows: the rectal swabs constituted the majority (87%) of samples at the baseline interval, but the availability of the stool samples progressively increased for larger proportions of patients (61% of patients at late interval; Fig. 1B).

**FIG 2:**
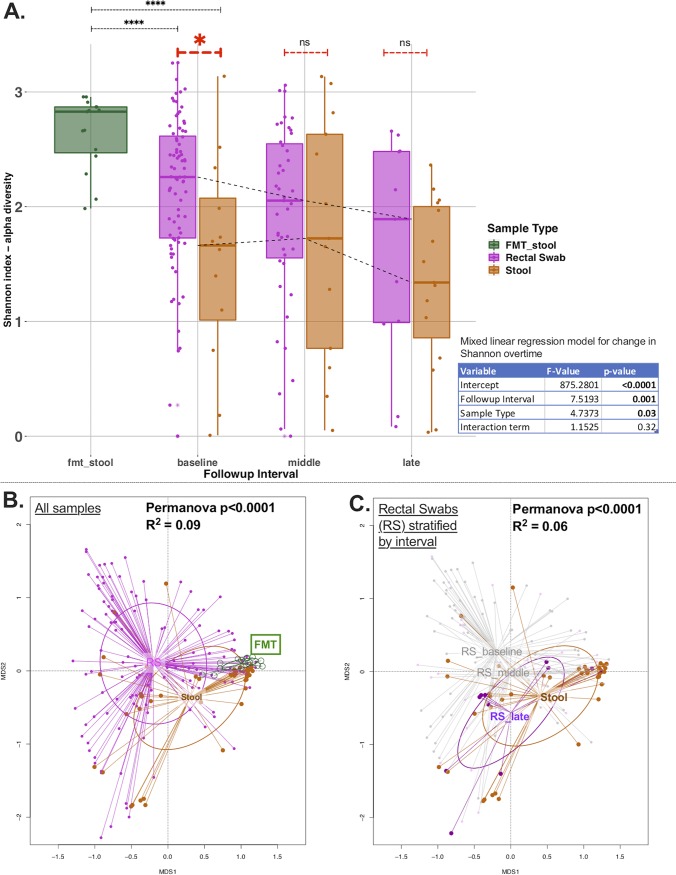
Alpha-diversity and beta-diversity comparisons show markedly different representations of the gut microbiome by sample type. (A) Alpha-diversity analyses by sample type and follow-up interval showed that rectal swabs had higher Shannon index values than stool samples at the baseline time point by a Wilcoxon test (*P* < 0.02) but not at subsequent follow-up intervals. Both rectal swabs and stool samples had significantly lower alpha-diversity than FMT samples (*P* < 0.0001) at baseline and at subsequent follow-up intervals. There was significant decline of Shannon index values over time, adjusting for sample type with a mixed linear regression model with random patient intercepts (shown in table inset). (B) Beta-diversity analyses: principal-coordinate analyses of Bray-Curtis dissimilarity indices between rectal swabs and stool samples. A greater distance between samples indicates greater dissimilarity. In the left panel, all available samples are stratified by sample type, showing significant differences between rectal swabs and stool samples (permutational multivariate analysis of variance [Permanova] *P* = 0.0001). FMT samples appeared compositionally more similar to stool samples than to rectal swabs from critically ill patients. In panel C, stratified analyses by study follow-up interval for rectal swabs show that rectal swabs in the late interval were more similar to stool samples (overlapping ellipsoids) than to rectal swabs obtained earlier (baseline or middle interval).

Rectal swabs and stool samples produced similar numbers of reads overall (high-quality 16S rRNA gene sequences; median [interquartile range {IQR}] = 4,235 [1,034]), which was a much higher level than the number of reads produced by experimental negative controls (Wilcoxon *P* < 0.0001; see Fig. S1 in the supplemental material), suggesting successful recovery of bacterial DNA signal by both sampling techniques. In the baseline interval, the rectal swabs had higher alpha-diversity values (median Shannon value = 2.3 [IQR value = 0.9]) than the stool samples (1.7 [1.1], Wilcoxon *P* = 0.02) (Fig. 2A; see also multivariate-adjusted analysis in Table S1 in the supplemental material), but samples collected later had similar alpha-diversity values (*P* = 0.68 and 0.30 for middle and late interval comparisons, respectively). Notably, both the rectal swabs and the stool samples had significantly lower alpha-diversity at baseline than the FMT stool samples from healthy donors (Wilcoxon *P* < 0.0001; Fig. 2A). Over time, there was a progressive decline in the alpha-diversity of ICU samples seen after adjusting for sample type (rectal swab versus stool samples), and these follow-up ICU samples continued to have lower alpha-diversity than the FMT stool samples (Fig. 2A; see also Fig. S2).

10.1128/mSphere.00358-19.2 FIG S1 Numbers of reads (high-quality 16S rRNA gene sequences) by sample type. Data from clinical samples are shown with colored filled box plots (green for stool samples from healthy donors of fecal microbiota transplant specimens, pink for rectal swabs from critically ill patients, and brown for stool samples from critically ill patients), whereas data from experimental controls are shown with empty box plots. Median and interquartile range (IQR) data corresponding to the number of reads for each sample type are shown with blue fonts. Comparisons of number of reads between samples were performed with nonparametric Wilcoxon tests. Gut microbiome specimens from critically ill patients (rectal swabs or stool samples) had numbers of reads in the range of PCR-positive controls (pcrpos) but had much higher numbers of reads than the negative controls (including negative controls for DNA extractions [mobioextrneg] and PCR-negative controls [pcrneg] as well as fecal microbiota transplant specimens [fmt_stool]). There were no differences in the median numbers of reads between rectal swabs (median, 4,270; IQR, 1,022) and stool samples (median, 4,185; IQR, 1,079) (*P* = 0.25). Download FIG S1, TIF file, 2.2 MB. Copyright © 2019 Fair et al. 2019 Fair et al. This content is distributed under the terms of the Creative Commons Attribution 4.0 International license.

10.1128/mSphere.00358-19.3 FIG S2 Significant decline in alpha-diversity (Shannon index) over time independently of sample type for the subset of patients (*n* = 27) with available samples at all follow-up intervals. No significant differences in Shannon index values were seen between rectal swabs and stool samples at each interval. There was significant decline of Shannon index values over time, adjusting for sample type with a mixed linear regression model with random patient intercepts (shown in the inset table). Download FIG S2, TIF file, 2.6 MB. Copyright © 2019 Fair et al. 2019 Fair et al. This content is distributed under the terms of the Creative Commons Attribution 4.0 International license.

10.1128/mSphere.00358-19.8 TABLE S1 Results of experiments using the mixed linear regression model for analysis of independent effects of sample type on baseline sample alpha-diversity (Shannon index). Statistically significant associations (*P* < 0.05) are shown in bold. Sample type was significantly associated with alpha-diversity after adjusting for potential confounders. Download Table S1, TIF file, 0.3 MB. Copyright © 2019 Fair et al. 2019 Fair et al. This content is distributed under the terms of the Creative Commons Attribution 4.0 International license.

Rectal swabs and stool samples were systematically different by beta-diversity data (Bray-Curtis dissimilarity index [permutational multivariate analysis of variance {PERMANOVA} *P* = <0.0001]; Fig. 2B), even after adjustment for potential confounders (Table S2). Of note, FMT stool samples were more similar to stool samples from ICU patients than to rectal swabs by beta-diversity analysis (Fig. 2B). By stratifying rectal swabs based on follow-up interval, visualization of beta-diversity data with principal-coordinate analyses (PCoA) revealed that the rectal swabs in the late interval were compositionally more similar to the stool samples than to the rectal swabs obtained earlier (Fig. 2C). By PERMANOVA, a statistically significant temporal effect for changes in beta-diversity values was found only for rectal swabs (*P* = 0.002) and not for stool samples. Next, in the subset of patients with both stool and rectal swabs available at different intervals, we examined the relative impact of patient identity versus the sample type variable on the beta-diversity data (effectively asking whether different sample types obtained from the same patient were more similar to each other than to same sample types obtained from different patients). By PERMANOVA, the sample type was the only variable significantly associated with differences in beta-diversity values (*P* = 0.002) (Table S3); i.e., knowing whether a community taxonomic profile was derived from a rectal swab or from a stool sample was more important than knowing from which patient the sample was taken. In further sensitivity analyses, we examined the potential impact of different methods of stool sample acquisition (collection from a rectal tube bag versus from bowel movements) but did not find any significant alpha-diversity or beta-diversity differences (Fig. S3).

10.1128/mSphere.00358-19.4 FIG S3 No significant alpha-diversity (A) or beta-diversity (B) differences were seen between stool samples obtained from patient bowel movements and liquid stool collected from rectal tube bags. Download FIG S3, TIF file, 2.8 MB. Copyright © 2019 Fair et al. 2019 Fair et al. This content is distributed under the terms of the Creative Commons Attribution 4.0 International license.

10.1128/mSphere.00358-19.9 TABLE S2 Significant independent associations of sample type with beta-diversity differences based on permutational multivariate analysis of variance models accounting for effects of potential confounders. Panel A includes results obtained with the model considering all samples available and panel B those obtained considering only baseline samples. Statistically significant associations (*P* < 0.05) are highlighted in bold. Download Table S2, TIF file, 0.5 MB. Copyright © 2019 Fair et al. 2019 Fair et al. This content is distributed under the terms of the Creative Commons Attribution 4.0 International license.

10.1128/mSphere.00358-19.10 TABLE S3 Permutational multivariate analysis of variance model for assessment of the impact of patient identity versus sample type on beta-diversity. The sample type variable was the only one significantly associated with beta-diversity differences (*P* < 0.0001); i.e., knowing whether a community taxonomic profile emerged from a rectal swab or from a stool sample was more important than knowing from which patient the sample was taken. Download Table S3, TIF file, 0.10 MB. Copyright © 2019 Fair et al. 2019 Fair et al. This content is distributed under the terms of the Creative Commons Attribution 4.0 International license.

Analyzing the baseline taxonomic composition at the phylum level, we noted that the rectal swabs had higher relative abundances of *Actinobacteria* (a commensal skin microbe) than the stool samples (Wilcoxon *P* = 0.05 for additive log ratio transformation comparison). *Actinobacteria* abundance declined significantly over time only in rectal swabs and not in stool samples (Fig. S4). At the genus level, stool samples had higher relative abundances of members of the *Akkermansia*, *Bacteroides*, *Enterococcus*, and *Parabacteroides* taxa, which are considered typical members of the gut microbiome in critically ill patients (Fig. S5). Examination of the taxonomic composition at the genus level for 10 patients who had both sample types available at different follow-up intervals showed marked discordance between the rectal swabs and the stool samples (Fig. S6).

10.1128/mSphere.00358-19.5 FIG S4 (A) Taxonomic composition at the phylum level for rectal swabs, stool samples, and FMT samples. Relative abundances of the 4 most common phyla (*Firmicutes*, *Bacteroidetes*, *Proteobacteria*, and *Actinobacteria*) are shown, broken down by follow-up interval for rectal swabs and stool samples. (B and C) Additive log-ratio-transformed abundances of *Actinobacteria* over time in rectal swabs (B) and stool samples (C). Significant declines in *Actinobacteria* abundance were evident for the rectal swabs only, examined by mixed linear regression model with study follow-up interval as an independent variable and additive log ratio of abundance as the response variable, with random patient intercepts. Download FIG S4, TIF file, 2.6 MB. Copyright © 2019 Fair et al. 2019 Fair et al. This content is distributed under the terms of the Creative Commons Attribution 4.0 International license.

10.1128/mSphere.00358-19.6 FIG S5 Box plot of relative abundances of taxa by sample type for the 15 most common taxa and other genera. Significantly increased relative abundances of the *Akkermansia*, *Bacteroides*, *Enterococcus*, and *Parabacteroides* taxa in stool samples versus rectal swabs are displayed. Comparisons were done with Wilcoxon tests on additive log ratio-transformed abundances of individual genera. Statistical significance is shown with asterisks (ns, not significant; *, *P* < 0.05; **, *P* < 0.01; ***, *P* < 0.001; ****, *P* < 0.0001). Download FIG S5, TIF file, 2.7 MB. Copyright © 2019 Fair et al. 2019 Fair et al. This content is distributed under the terms of the Creative Commons Attribution 4.0 International license.

10.1128/mSphere.00358-19.7 FIG S6 Taxonomic composition bar plot at the genus level for 10 patients with both stool samples and rectal swabs available at different time points. Rectal swabs are shown in the top row and stool samples in the bottom row, with each patient’s unique 4-digit identifier (ID) highlighting samples belonging to the same patient. The relative abundances of the 15 most common taxa are shown with different colors, and the additional relative abundances of all other component taxa are shown in gray (“other genera”). The data demonstrate marked discordance between the taxonomic composition of rectal swabs and that of stool samples. Download FIG S6, TIF file, 2.8 MB. Copyright © 2019 Fair et al. 2019 Fair et al. This content is distributed under the terms of the Creative Commons Attribution 4.0 International license.

Our analyses in a large cohort of critically ill patients highlight significant differences between the more accessible rectal swabs and the harder to obtain, but commonly viewed as reference standard stool samples. In our study, stool sample availability captured distinct patient characteristics, perhaps because morbid critically ill patients with longer ICU stays had higher likelihood of stool passage and collection during the study follow-up period. Nevertheless, stool samples and rectal swabs had significant differences in alpha and beta-diversity even after adjustment for potential confounders of the associations between sample types and microbiota community composition.

Systematic differences in alpha-diversity and beta-diversity by sample type were largely attributable to the baseline samples acquired at time points close to ICU admission. At these early time points, 87% of the available samples were rectal swabs and most (58%) of the patients were not receiving enteral nutrition. Stool presence in the rectal vault may have been limited, leading to “unsoiled” swabbing of the rectal mucosa and perirectal skin, with a resulting disproportionately higher abundance of skin bacteria (i.e., members of the *Actinobacteria* phyla) than would normally be expected for gut microbiota profiles (11). The temporal convergence of microbial profiles between rectal swabs and stool samples observed in our study suggests that progressive recovery of gut motility during the ICU course and stool presence in the rectal vault may have improved the reliability of “soiled” rectal swab sampling, although we did not qualitatively score the macroscopic appearance of swabs as performed previously in the study by Bansal et al. (9). In addition, our study design with periodic sampling in predefined intervals rather than on consecutive days hindered our ability to detect day-to-day dynamic changes of gut microbiota communities and limited the number of follow-up samples in our cohort. Thus, analyses in the late interval have low statistical power and may have also been affected by informative censoring, i.e., patients not contributing late follow-up samples due to rapid clinical improvement and discharge from the ICU or due to clinical deterioration and early ICU death. Since we were not able to perform head-to-head comparisons of rectal swabs and stool samples obtained at the same time, the notable discordance of microbial community profiles by sample type observed in the small subset of patients with longitudinal samples of both types requires cautious interpretation, given that it was not possible to account for patient-level temporal variability.

Our results call for caution in the design of gut microbiome studies in critically ill patients. Despite their availability, rectal swabs may offer biased representations of the presumed gut microbial communities, especially when sampling is conducted early in the course of critical illness. Consequently, analyses of gut microbiota studies need to take into account both the actual sample types used and the timing of sample acquisition, because rectal swabs and stool samples are not interchangeable for the purpose of microbiota sequencing profiling. Accurate and reproducible delineation of the role of the gut microbiome in critical illness will require consistent sampling protocols, recording of clinical variables, and longitudinal assessments.

### Data availability.

Raw sequences used for this project have been deposited in the BioProject database and are publicly available at https://www.ncbi.nlm.nih.gov/bioproject/516701. Taxon tables, metadata, and the R statistical code required to conduct the analyses described here are publicly available at https://github.com/MicrobiomeALIR/.
