# Supplementary material for: Rectal Swabs from Critically Ill Patients Provide Discordant Representations of the Gut Microbiome Compared to Stool Samples
Source: mSphere. 2019 Jul 24;4(4):e00358-19. doi: 10.1128/mSphere.00358-19 (PMC6656869; doi:10.1128/mSphere.00358-19)
Supplement: Text S1 [file mSphere.00358-19-s0001.pdf]

Rectal swabs in critically ill patients provide discordant representations of the gut microbiome compared to stool samples

Supplemental Methods

Contents:

Expanded Methods: pages 2-9

References: pages 10-11

Expanded Methods

Clinical cohort:

From March 2015 – August 2017, we prospectively enrolled consecutive mechanically-ventilated patients with acute respiratory failure from the Medical Intensive Care Unit (ICU) at the University of Pittsburgh Medical Center (UPMC) into the Pittsburgh Acute Lung Injury Registry and Biospecimen Repository (ALIR). Eligible patients were 18 years or older with acute respiratory failure who were intubated and mechanically-ventilated. Exclusion criteria included inability to obtain informed consent, presence of tracheostomy, or mechanical ventilation for more than 72 hours prior to enrollment. The ALIR study was approved by the University of Pittsburgh Institutional Review Board (protocol PRO10110387), and written informed consent was provided by all participants or their surrogates in accordance with the Declaration of Helsinki.

For comparisons with healthy controls, we also included samples from donated stool for fecal microbiota transplant from 15 healthy adult donors.

Sample Collection:

From consented critically-ill patients, we collected rectal swabs and/or stool samples at three time intervals starting at the time of intubation and continuing up to 10 days if the patient remained in the ICU (defined as baseline [days 0-2], middle [days 3-6] and late intervals of follow-up [days 7-10]). Rectal swabs were collected as per routine clinical practices (i.e. placing the patient in a lateral position, entering the tip of the 6" cotton tip swab in the rectal canal and rotating gently for 5 seconds), unless clinical reasons precluded movement of the patient (e.g. severe hemodynamic or respiratory instability). Stool samples were collected when available,

either by taking a small sample from an expelled bowel movement (before cleaning of the patient and disposal of stool), or through a fecal management system (rectal tube) placed for management of diarrhea and liquid stool collection.

Stool samples or rectal swabs were placed in sterile collection tubes and then capped, labeled and stored at -80°C until further processing.

Clinical Data Recording:

Detailed data was obtained from the electronic medical record on the day of enrollment including:

1) Demographic information: age, sex, height, weight, body mass index
2) Laboratory results: white blood cell count, hemoglobin, platelets, serum bicarbonate, arterial pH, partial pressure of oxygen, partial pressure of carbon dioxide.
3) Mechanical ventilation parameters: respiratory rate, tidal volume (normalized per kilogram ideal body weight), positive end-expiratory pressure, peak inspiratory pressure, and fraction inspired oxygen concentration.
4) Severity of illness scores: sequential organ failure assessment (SOFA) score excluding the neurologic component because all patients were intubated and receiving sedative medications, impairing our ability to perform assessment of the Glasgow Coma Scale in a consistent and reproducible fashion) (1). SOFA score was calculated by using the physiologically worse values within 24hrs of enrollment.
5) Lung Injury Prediction Scores (2).
6) Medication administration: antibiotics, vasopressors, and sedatives administered during the first week of ICU course from intubation.

7) Gastrointestinal/Nutritional Factors: enteral nutrition administration, number of bowel movements or clinical diarrhea (as assessed by bedside nurses), and rectal tube presence.
8) Outcomes: length of ICU stay, 30-day mortality, incident shock, sepsis, and occurrence of acute kidney injury (AKI) within the first week of ICU stay(4).

Laboratory Methods:

DNA extraction:

We extracted bacterial DNA directly from samples (rectal swabs and stool samples) using Powersoil (Qiagen) extraction kit following manufacturer's instructions, as previously described (3). For rectal swabs, we agitated the cotton tip in the extraction column tube for e1-2 min and until visible particles of the of biospecimen were released from the cotton tip into the C1 solution of the Powersoil kit. For stool samples, we used 30-40mg of stool sample directly inoculated into the C1 solution of the extraction column tube. As DNA extraction negative controls, we added DNA-free sterile water in one extraction column per each batch of clinical sample DNA extractions (~12-15 samples per batch).

16S rRNA gene sequencing:

We amplified extracted DNA by PCR using the method of Caporaso et al. and the Q5 HS High-Fidelity polymerase (NEB) targeting the V4 hypervariable region of the 16S rRNA gene (5). The exact primer sequences were the following:

- 515F: GTGCCAGCMGCCGCGGTAA
- 806R: GGACTACHVGGGTWTCTAAT

Degeneracy was introduced with the following ambiguous bases:

M: A or C

H: A or C or T

V: A or C or G

W: A or T

We utilized reagent controls for each step of the process (DNA extraction and PCR amplification as described above). We amplified four microliters per reaction of each sample with a single barcode in triplicate 25 microliter reactions. As PCR-negative controls, we added DNA-free sterile water in the PCR reaction mix in amount equal to the amount of template DNA from clinical samples used (typically 6 microliters). As PCR amplification positive controls, we utilized the ZymoBIOMICs Microbial Community DNA Standard (Zymo Research, Irvine, CA), a mock microbial community consisting of genomic DNA of eight bacterial strains. We utilized 1 microliter of the genomic mixture with concentration of 10ng/microliter for each reaction. Cycle conditions were 98˚C for 30s, then 33 cycles of 98˚C for 10s, 57˚C for 30s, 72˚C for 30s, with a final extension step of 72˚C for 2 min. We combined triplicates and purified with the AMPure XP beads (Beckman) at a 0.8:1 ratio (beads:DNA) to remove primer-dimers. We quantitated eluted DNA on a Qubit fluorimeter (Life Technologies). We performed sample pooling on ice by combining 20 ng of each purified band. For negative controls and poorly performing samples, we used 20 microliters of each sample. We purified the sample pool with the MinElute PCR purification kit. The final sample pool underwent two more purifications – AMPure XP beads to 0.8:1 to remove all traces of primer dimers and a final cleanup in Purelink

PCR Purification Kit (Life Technologies). We quantitated the purified pool in triplicate on the Qubit fluorimeter prior to preparing for sequencing. We prepared the sequencing pool according to instructions by Illumina, with an added incubation at 95˚C for 2 minutes immediately following the initial dilution to 20 picomolar. We then diluted the sequencing pool to a final concentration of 7 pM + 15% PhiX control. Amplicons were sequenced on the Miseq platform.

Analytics:

16S Sequence quality control:

Sequences from the pooled sequencing run were demultiplexed into individual sample/replicate fastq files. Each fastq file was then processed through the Center for Medicine and the Microbiome (CMM) custom modular read QC pipeline that was configured to perform the following steps: low complexity filtering, QV trimming, Illumina sequencing adapter trimming, and 16S primer trimming. Low complexity filtering utilized NCBI BLAST's dustmasker (6). Reads with greater than 80% low complexity regions were filtered. QV trimming and filtering utilized the FASTX Toolkit. The trimming threshold was QV>25 from the 3' end, and length>125 bp after trimming. The subsequent filtering threshold was >25 across >95% of the read. The Illumina sequencing adapter and 16S primer trimming was performed with cutadapt (7).

16S Clustering and annotation**:**

Paired sequences with forward and reverse reads passing the QC filtering and trimming steps were then mated (end aligned and a consensus sequence computed) using the make.contigs function of Mothur. Consensus sequences were screened to limit the overlap mismatch to no more than 20%. The maximum number of N's allowed in the overlap was 4 and the minimum

overlap was required to be greater than 25bp. Consensus sequences passing screening were then passed through CMM's 16S clustering and annotation pipeline, a Mothur-dependent wrapper designed to streamline and automate the execution of the following Mothur steps in version v.1.39.1: unique.seqs, align.seqs, screen.seqs, filter.seqs, second uniq.seqs, pre.cluster, chimera.uchime, remove.seqs, classify.seqs, dist.seqs, cluster, make.shared, and classify.otu. Mothur output files were then reformatted to sample x category (taxonomic levels or operational taxonomic units [OTU] at 97% sequence similarity). For downstream statistical modeling and analyses, we utilized taxonomic tables at the genus level, with taxonomic assignments performed using a naïve Bayes k-mer classifier in conjunction with the Ribosomal Database Project (RDP) 16S rRNA gene sequences (8).

Taxa table edits:

The taxa table was filtered for low abundance taxa (relative abundance, <0.005%) and singletons. We did not filter clinical samples for any taxa detected in the negative control samples. Five samples were subjected to repeat PCR amplification due to poor performance in initial reactions, and were then removed from analyses as duplicates. Samples with very few reads (<200) were excluded from analyses (n=5).

Statistical Analysis

We calculated descriptive statistics of baseline characteristics and performed non-parametric comparisons (Wilcoxon test for continuous and Fisher's exact test for categorical variables) using the R software (R Foundation for Statistical Computing, 2016). We performed ecological analyses of alpha-diversity (richness-Shannon), beta diversity (Bray–Curtis

dissimilarity index), and taxonomic relative abundance at the phylum and genus level with R *vegan* package (9). Beta-diversity comparisons with permutation analysis of variance (Permanova at 1000 permutations) were visualized with Principal Coordinates Analyses. Taxonomic abundance differences between groups were calculated following transformation of abundance data with the additive log ratio, performed for the top 4 phyla (Firmicutes, Bacteroidetes, Actinobacteria and Proteobacteria) and the top 20 genera (10).

In order to assess for longitudinal changes of alpha-diversity over time as well as to account for the effects of potential confounders on the associations between sample type and gut microbiota profiles, we constructed a set of multivariate models as described below.

As confounders, we considered potential variables that may impact gut motility and bowel content in critically-ill patients, as well as having potential associations with the gut microbiome:

1. Early Enteral Nutrition (defined as initiation of gastric or enteral feeds within 48hrs of ICU admission).
2. SOFA score on continuous scale, as a marker of severity of illness.
3. Presence of rectal tube, as an indicator of severe diarrhea requiring a fecal management system.
4. Obesity, defined as a BMI>30, which has been associated with gut microbiome profiles, but for practical purposes may also affect our ability to perform rectal swabbing especially in the subset of patients with morbid obesity.
5. Clinical diagnosis of sepsis, associated with administration of broad-spectrum antibiotics, which can cause diarrhea, but may also affect the gut microbial communities.

We then constructed the following multivariate models:

1. Alpha-diversity in baseline samples: we constructed a mixed linear regression model with Shannon index as the response variable, random patient intercepts, and then the sample type as an independent variable adjusted for these 5 confounders.
2. Alpha-diversity over time: we constructed mixed linear regression models considering random patient-level intercepts and examined for the independent effects of ordered follow-up intervals (baseline vs. middle vs. late) after adjusting for sample type (rectal swab vs. stool samples) for all patients as well as for the subset of patients with available follow-up samples (i.e. excluding patients with baseline samples only).
3. Beta-diversity: we performed PERMANOVA analyses with beta-diversity as the dependent variable and the sample type as an independent variable adjusted for the 5 confounding variables described above.

(1) Vincent JL, de Mendonça A, Cantraine F, Moreno R, Takala J, Suter PM, Sprung CL, Colardyn F, Blecher S. Use of the SOFA score to assess the incidence of organ dysfunction/failure in intensive care units: results of a multicenter, prospective study. Working group on "sepsis-related problems" of the European Society of Intensive Care Medicine. Crit Care Med 1998; 26 (11): 1793-800. PMID 9824069.

(2) Gajic O, Dabbagh O, Park PK, Adesanya A, Chang SY, Hou P, Anderson H 3rd, Hoth JJ, Mikkelsen ME, Gentile NT, Gong MN, Talmor D, Bajwa E, Watkins TR, Festic E, Yilmaz M, Iscimen R, Kaufman Da, Esper AM, Sadikot R, Douglas I, Sevransky J, Malinchoc M, US Critical Illness and Injury Trials Group: Lung Injury Prevention Study Investigators (USCIITG-LIPS). Am J Respir Crit Care Med 2011; 183 (4): 462-70. doi: 10.1164/rccm.201004-0549OC.

(3) Singer M, Deutschman CS, Seymour CW, Shankar-Hari M, Annane D, Bauer M, Bellomo R, Bernard GR, Chiche JD, Coopersmith CM, Hotchkiss RS, Levy MM, Marshall JC, Martin GS, Opal SM, Rubenfeld GD, van de Poll T, Vincent JL, and Angus DC. The Third International Consensus Definitions for Sepsis and Septic Shock (Sepsis-3). JAMA 2016; 315: 801–810. doi:10.1001/jama.2016.0287.

(4) Morris A, Beck JM, Schloss PD, Campbell TB, Crothers K, Curtis JL, Flores SC, Fontenot AP, Ghedin E, Huang L, Jablonski K, Kleerup E, Lynch SV, Sodergren E, Twigg H, Young VB, Bassis CM, Venjataraman A, Schmidt TM, Weinstock GM, and Lung HIV Microbiome Project. Comparison of the respiratory microbiome in healthy nonsmokers and smokers. Am J Respir Crit Care Med 2013; 187: 1067–1075. doi:10.1164/rccm.201210-1913OC.

(5) Caporaso JG, Lauber CL, Walters WA, Berg-Lyons D, Huntley J, Fierer N, Owens SM, Betley J, Fraser, Bauer M, Gormley N, Gilbert JA, Smith G, Knight R. Ultra-high-throughput microbial community analysis on the Illumina HiSeq and MiSeq platforms. ISME 2012; 6(8):1621–1624. doi:10.1038/ismej.2012.8.

(6) Morgulis A, Gertz EM, Schäffer AA, and Agarwala R. A fast and symmetric DUST implementation to mask low-complexity DNA sequences. J Comput Biol 2006; 13: 1028–1040. doi:10.1089/cmb.2006.13.1028.

(7) Haas B J, Gevers D, Earl AM, Feldgarden M, Ward DV, Giannoukos G, Ciulla D, Tabbaa D, Highlander SK, Sodergren E, Methé B, DeSantis TZ, Human Microbiome Consortium, Petrosino JF, Knight R, Birren BW. Chimeric 16S rRNA sequence formation and detection in Sanger and 454-pyrosequenced PCR amplicons. Genome Res 2011; 21: 494–504. doi:10.1101/gr.112730.110.

(8) Schloss PD, Westcott SL, Ryabin T, Hall JR, Hartmann M, Hollister EB, Lesniewski RA, Oakley BB, Parks DH, Robinson CJ, Sahl JW, Stres B, Thallinger GG, Van Horn DJ, and Weber CF. Introducing mothur: open-source, platform-independent, community-supported software for describing and comparing microbial communities. Appl Environ Microbiol 2009; 75: 7537–7541. doi:10.1128/AEM.01541-09.

(9) Dixon P. VEGAN, a package of R functions for community ecology. Journal of Vegetation Science 2003. 14: 927–930. doi:10.1111/j.1654-1103.2003.tb02228.x.

(10) Tsilimigras MCB, Fodor AA. Compositional data analysis of the microbiome: fundamentals, tools, and challenges. Annals of Epidemiology 2016; 26(5): 330-335. doi:10.1016/j.annepidem.2016.03.002.
