# Supplementary figures and images for: Rectal Swabs from Critically Ill Patients Provide Discordant Representations of the Gut Microbiome Compared to Stool Samples
Source: mSphere. 2019 Jul 24;4(4):e00358-19. doi: 10.1128/mSphere.00358-19 (PMC6656869; doi:10.1128/mSphere.00358-19)

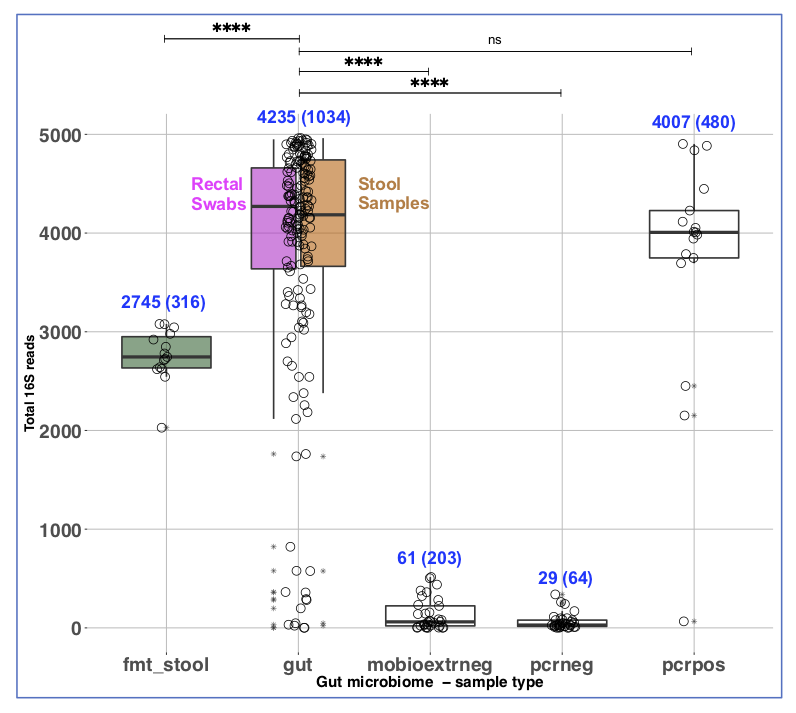

Supplement: FIG S1 [file mSphere.00358-19-sf001.tif]

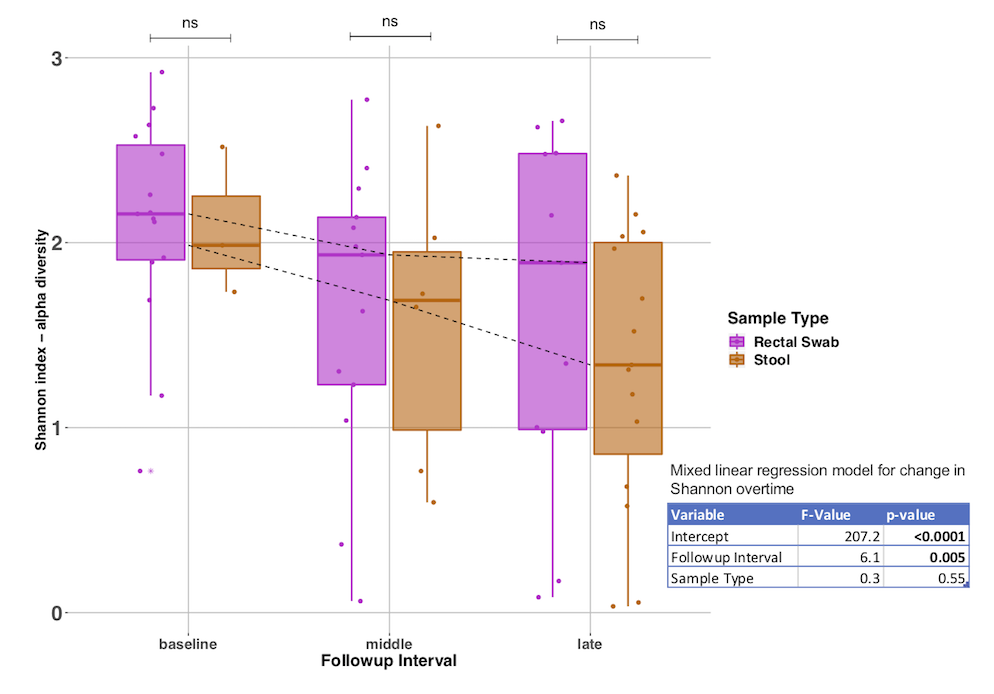

Supplement: FIG S2 [file mSphere.00358-19-sf002.tif]

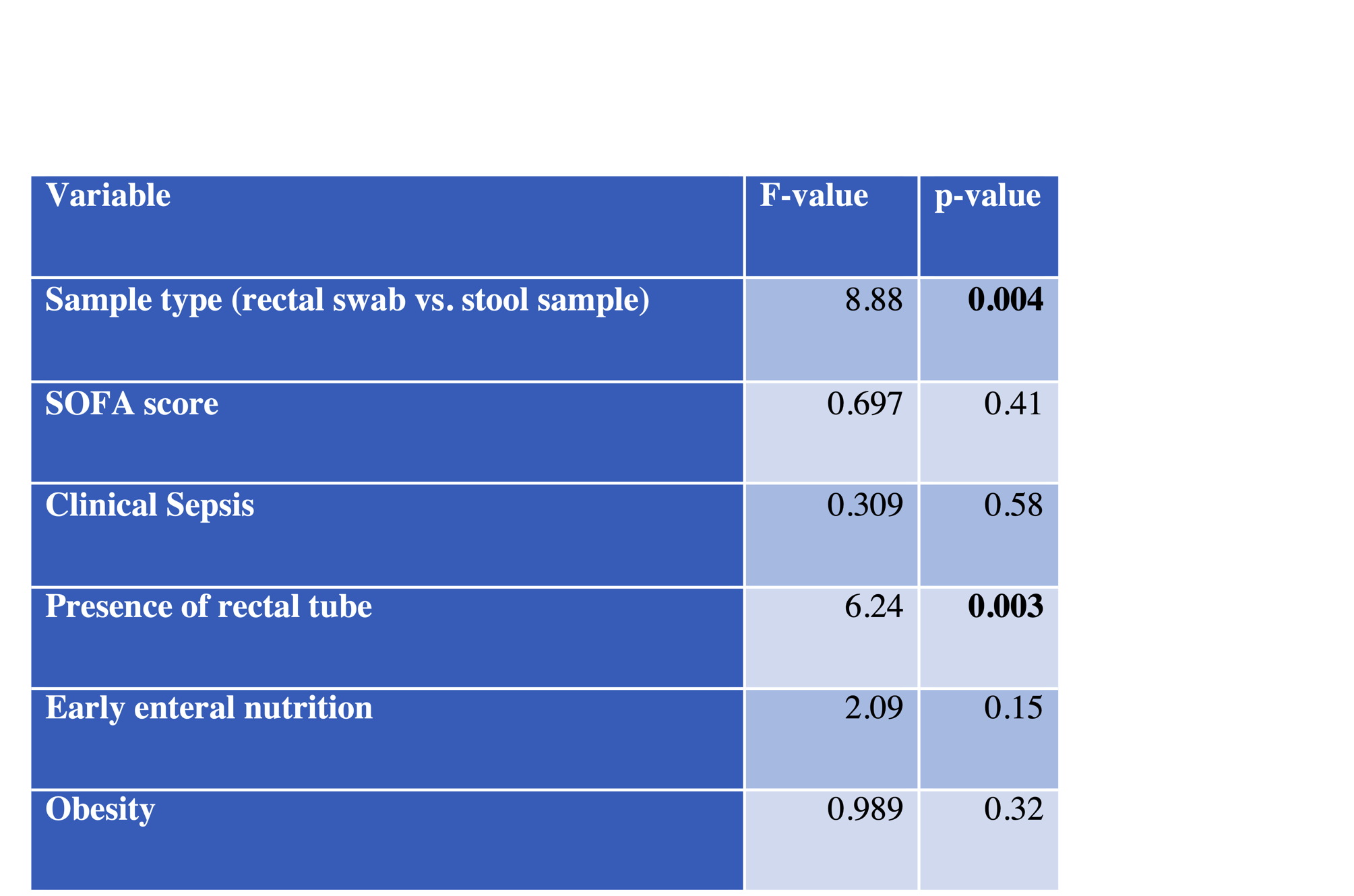

Supplement: TABLE S1 [file mSphere.00358-19-st001.tif]

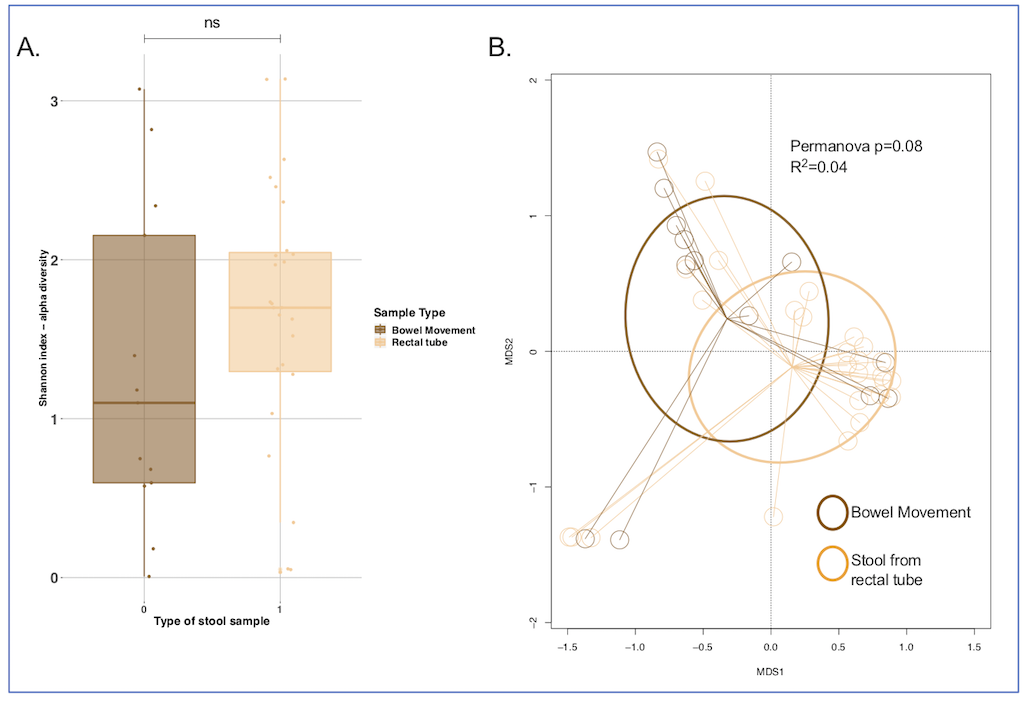

Supplement: FIG S3 [file mSphere.00358-19-sf003.tif]

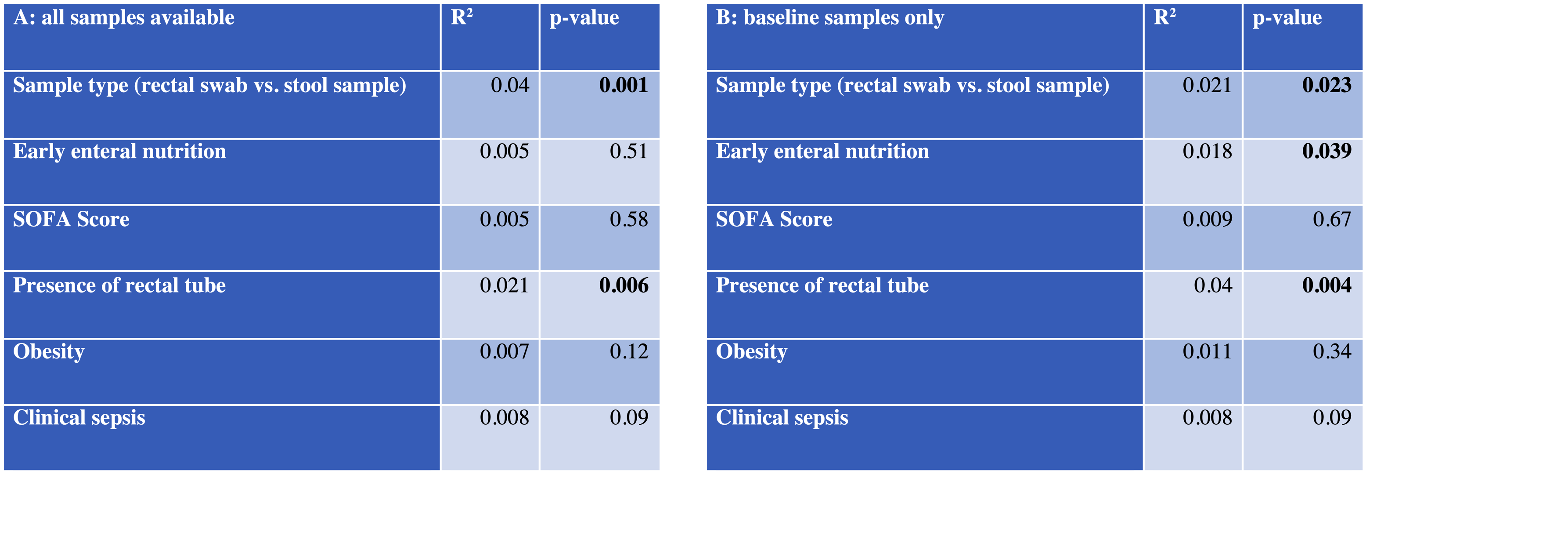

Supplement: TABLE S2 [file mSphere.00358-19-st002.tif]

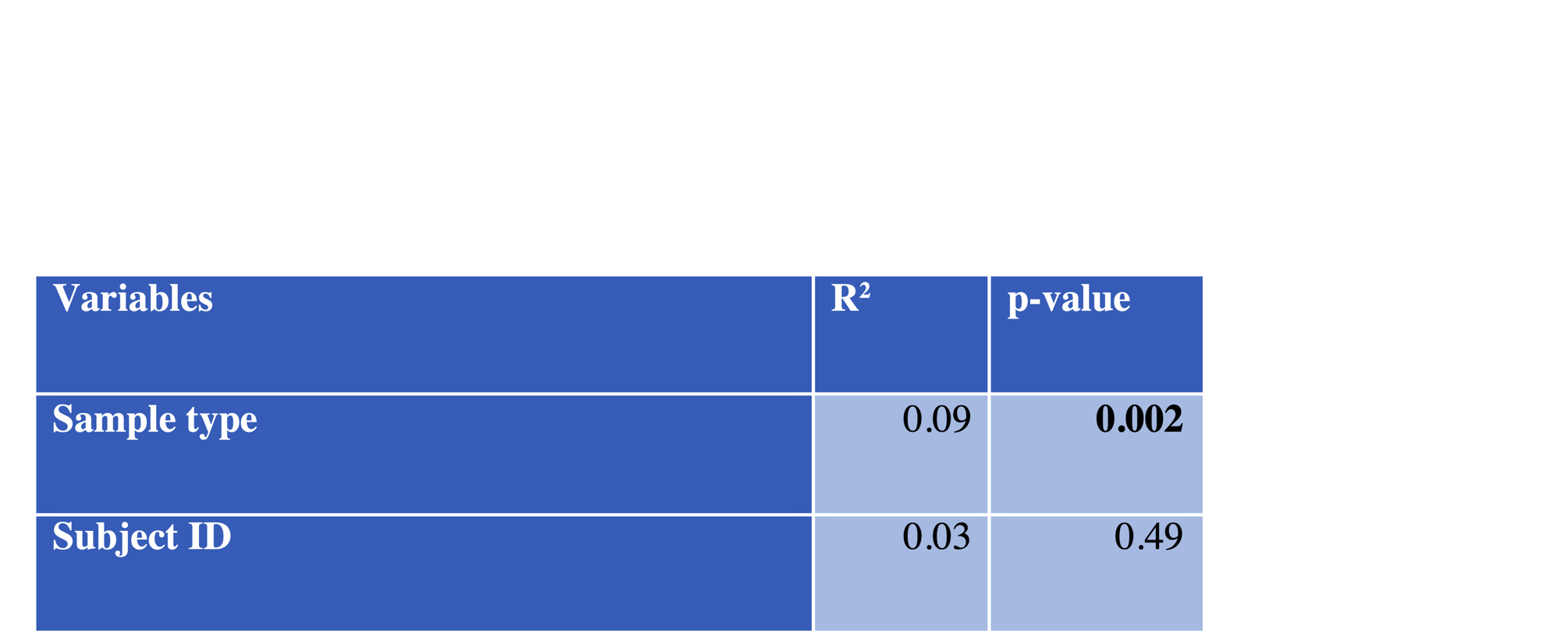

Supplement: TABLE S3 [file mSphere.00358-19-st003.tif]

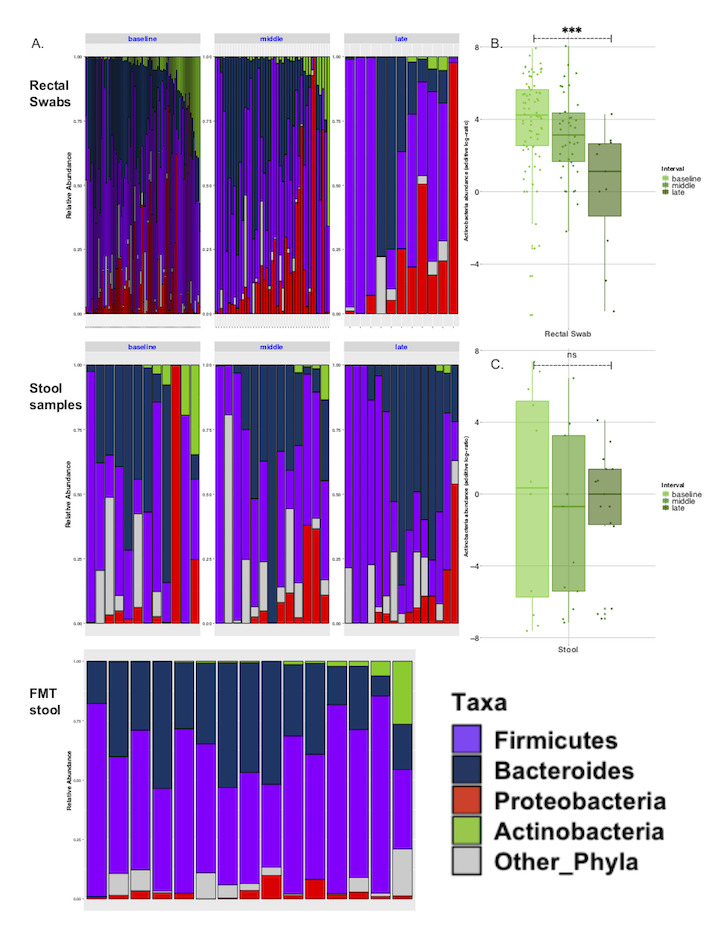

Supplement: FIG S4 [file mSphere.00358-19-sf004.tif]

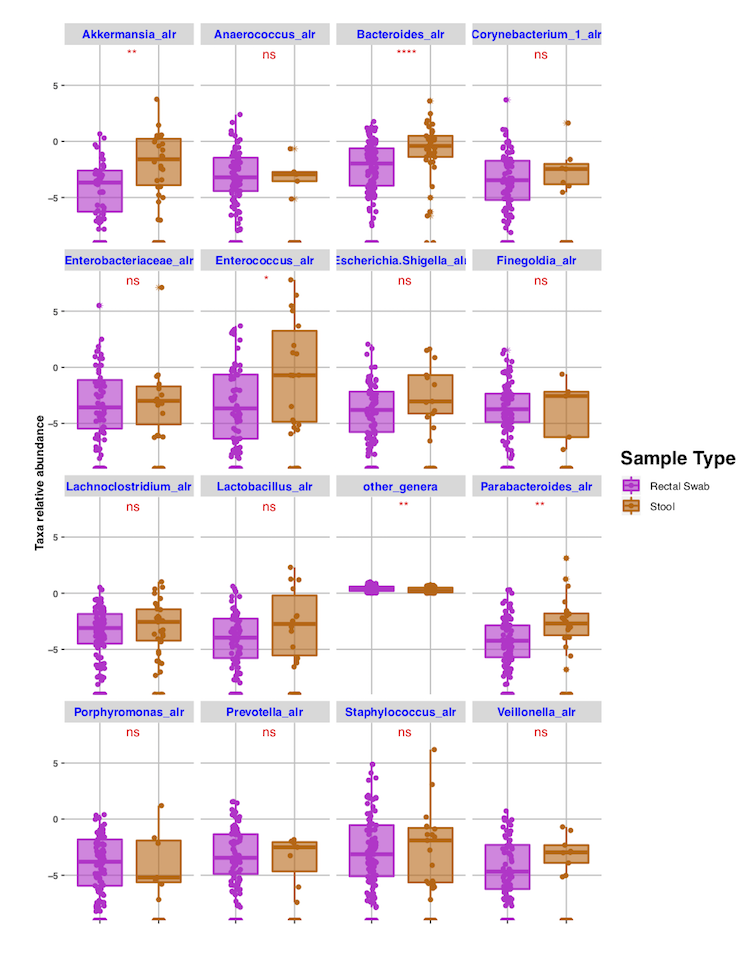

Supplement: FIG S5 [file mSphere.00358-19-sf005.tif]

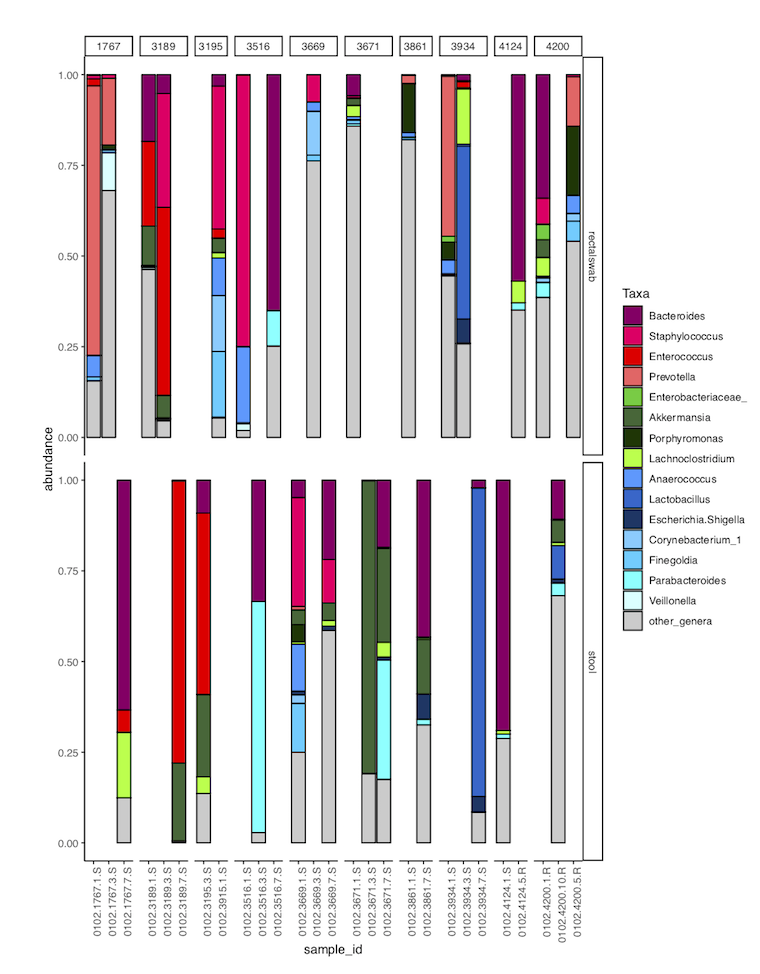

Supplement: FIG S6 [file mSphere.00358-19-sf006.tif]
